# Elements of the Branches of Natural Philosophy Connected with Medicine; Viz.Chemistry, Optics, Acoustics, Hydrostatics, Electricity, and Physiology; Including the Doctrine of the Atmosphere, Fire, Phlogiston, Water, &c. Together with Bergman's Tables of Electrive Attractions, with Explanations and Improvements

**Published:** 1782

**Authors:** 


VII. *Elements of the Branches of Natural Philoſophy connected with Medicine; viz. Chemiſtry, Optics, Acouſtics, Hydroſtatics, Electricity, and Phyſiology; including the Doctrine of the Atmoſphere, Fire, Phlogiſton, Water, &c. Together with Bergman's Tables of Electrive Attractions, with Explanations and Improvements. By* J. Elliott, M. D. 8vo. Johnſon. *London.* 1782. 302 pages with 4 copper-plates.

THE utility of natural philoſophy to the medical practitioner, muſt be ſufficiently obvious, when it is conſidered that the faculties of of the human body are intimately connected with thofe powers of nature which are in a more efpecial manner the object of that fcience. But for want of fome eafy compendium like the prefent, this interefting branch of knowledge has perhaps been lefs generally cultivated than it ought to be by ftudents of phyfic.

The whole is written in a clear familiar manner, and is divided into three parts. The firft treats of chemiftry, including the doctrine of the atmofphere, fire, and other fubjects connected with that fcience. The fecond relates to optics, acouftics, hydroftatics, and electricity; neither of which could properly be comprifed under the head of chemiftry. The third is confined to phyfiology, or the philofophy of the human body.

To give a minute account of a performance of this nature would be fuperfluous, we fhall therefore prefent our readers with the following extract from the fection on chemical elective attractions, as a fpecimen of the author's manner.

" Spirit of vitriol is a combination of vitriolic acid with water. If to this compound you add thin plates of copper, and give a due degree of heat, the copper will be diffolved. The mixture ture therefore will be blue vitriol and water, which might easily be separated by the methods already described. If to this solution you add plates of iron, the acid will leave the copper, and unite with the iron. As the latter dissolves, and the former is let go by the acid, it deposits itself upon the iron plates, so that they look like copper: but when the iron is all dissolved, the copper will fall to the bottom in form of a powder. The clear liquid being decanted will therefore be a mixture of water and green vitriol. If to this solution zinc be added, the acid will leave the iron by degrees, and unite with the zinc, and the iron will fall to the bottom in form of a powder, as the copper did before. The clear liquor being decanted, will be a solution of white vitriol in water. Add volatile alcali to this solution, the acid will leave the zinc to unite with the salt; the zinc will be precipitated, and the clear liquor being decanted will be vitriolic sal ammoniac and water. Add *fixed* alcali to this liquid, the acid will unite with it, letting go the *volatile* and the mixture will be vitriolated tartar, and spirit of sal ammoniac. The latter may be obtained by distillation, the former remaining behind. Mix this salt with an equal quantity of fixed fixed alcali, and add powdered charcoal, equal to about a fourth part of the weight of the whole. The charcoal, you will obſerve, contains phlogiſton, combined with a vegetable earth, and the alcali is added to make the vitriolated tartar melt, which it will not eaſily do without ſuch addition. Put theſe ingredients into a crucible, and apply a ſudden and ſtrong heat for a ſhort time; the vitriolic acid will leave the fixed alcali, to unite with the phlogiſton of the charcoal. The mixture therefore is now common ſulphur, fixed alcali, and vegetable earth; that is, an impure liver of ſulphur. Diſſolve the maſs in water, the earth will ſubſide, and the clear liquid will be a ſolution of liver of ſulphur, and fixed alcali in water. Decant or filter this liquid; and to obtain the ſulphur, look in the table for fixed alcali; you will find above ſulphur ſeveral acids. Add a ſufficient quantity of either of theſe, the alcali will quit the ſulphur to unite with the acid, and the ſulphur will fall to the bottom in a powder, which you may ſeparate from the liquid, and melt into a roll.—And thus will you have had ſucceſſively ſpirit of vitriol, blue vitriol, green vitriol, white vitriol, vitriolic ſal ammoniac, vitriolated tartar, and

ſulphur;

ſulphur; each of which might eaſily have been obtained in their uſual forms."

